# Change in Thermodynamic Entropy and Free Volume of Epoxy Resin During Tensile Deformation

**DOI:** 10.3390/polym17040477

**Published:** 2025-02-12

**Authors:** Takuma Inoue, Yutaka Oya, Jun Koyanagi, Takenobu Sakai

**Affiliations:** 1 Graduate School of Science and Engineering, Saitama University, Saitama 338-8570, Japan; 2 Research Institute for Science and Technology, Tokyo University of Science, Tokyo 125-8585, Japan; oya@rs.tus.ac.jp; 3 Department of Materials Science and Technology, Tokyo University of Science, Tokyo 125-8585, Japan; koyanagi@rs.tus.ac.jp

**Keywords:** thermodynamic entropy generation, specific heat capacity, differential scanning calorimetry, free volume, positron annihilation lifetime spectroscopy, volumetric strain, epoxy resin

## Abstract

The relationship between thermodynamic entropy generation and free volume changes during the tensile deformation of epoxy resin was investigated. Thermodynamic entropy generation was evaluated using differential scanning calorimetry (DSC) for samples at various strain levels, while free volume changes were measured with positron annihilation lifetime spectroscopy (PALS). Volumetric strain was assessed through the digital image correlation (DIC) method. The results showed that both thermodynamic entropy and free volume increase during tensile deformation, and the average free volume radius becomes more uniform. It was observed that thermodynamic entropy generation and free volume each exhibit a linear relationship with volumetric strain. Additionally, thermodynamic entropy generation increased linearly with free volume. These findings suggest that the increase in thermodynamic entropy during tensile deformation is attributed to irreversible changes, such as the expansion of free volume within the material.

## 1. Introduction

In recent years, growing awareness of global warming and environmental pollution has accelerated the adoption of carbon-fiber-reinforced plastics (CFRPs) in industries such as aerospace and the automotive industry. This acceleration is driven by the need to reduce energy consumption and CO_2_ emissions through lightweight structural design [1]. CFRP offers significant advantages, including high specific strength, high rigidity, and excellent corrosion resistance. However, compared to metallic materials, CFRP presents challenges, such as low recyclability and high energy consumption during the manufacturing process [2,3,4,5]. To minimize the environmental impact and contribute to global warming mitigation, it is essential to reduce the reliance on the mass production and consumption of these composite materials while emphasizing their long-term utilization. One potential solution to these challenges is to assess the remaining life of materials by evaluating their degradation status during use. The ability to quantitatively evaluate the remaining life of materials is expected not only to enhance reliability but also to reduce unnecessary periodic replacement. This would contribute to minimizing the mass production and consumption of materials. Current methods for assessing remaining life include accelerated degradation tests in high-temperature environments, as well as estimation techniques for polymer materials based on linear and nonlinear viscoelastic constitutive equations and time–temperature superposition principles [6,7,8,9]. However, accelerated degradation tests evaluate the remaining life when the material is not in use, making it difficult to predict its life under actual service conditions. In addition, applying TTSP-based life prediction methods to in-use materials is challenging, as it requires estimating the mechanical and thermal load history applied to the material. Therefore, to understand the remaining life of materials in use, it is essential to identify irreversible changes. To date, several studies have focused on increases in free volume and entropy generation as changes associated with the deformation of polymer materials. Some researchers have investigated the relationship between the free volume of polymers and their tensile strength and Young’s modulus [10,11,12,13,14,15]. It has been demonstrated that free volume significantly affects the mechanical properties of polymers. Therefore, evaluating changes in free volume due to deformation is crucial.

Bowman et al. [16] investigated structural and free volume changes during the creep of amorphous polyethylene under various stress states using all-atom dynamics simulations. They found that the evolution of free volume formation, growth, and coalescence was directly correlated with chain dynamics at each stage of the creep response, across all stress states and temperatures. Takase et al. [17] used all-atom MD simulations to examine the change in the number and size of voids in polyamide 6 (PA6) due to tensile deformation and found that the number of voids increases with increasing strain. Furthermore, Iwamoto et al. [18] demonstrated that the void volume fraction of polyetherketone (PEEK) increases with the number of fatigue cycles. Makarewicz et al. [19,20] and Huang et al. [21] investigated changes in free volume during the tensile deformation of thermoplastic resins, such as polypropylene and polyethylene, using small-angle X-ray scattering and positron annihilation lifetime spectroscopy (PALS). Their studies clarified that both the ratio and the shape of free volume change with tensile deformation. Additionally, Uedono et al. [22] performed PALS measurements on carbon-fiber-reinforced plastics after fracture and examined differences in the free volume radius depending on the position of the test specimen. Their results revealed that the free volume radius increased in the fractured region and that the free volume changes were associated with the scission and aggregation of molecular chains.

In recent years, a fracture criterion based on the irreversible entropy generated when a material is subjected to load or deformation has been proposed [23,24,25,26,27,28,29,30,31,32,33,34,35,36,37,38]. Naderi et al. [23] showed that fracture occurs when the entropy generation within a material reaches a certain threshold value.

This failure criterion is often based on entropy generation arising from the plastic strain energy stored in the material and the dissipated energy. These are referred to as fatigue fracture entropy (FFE) and mechanical entropy. In many studies, mechanical entropy ($\Delta \gamma_{M}$) is expressed by the following equation:

(1) $$\Delta \gamma_{M}=\int_{0}^{t_{f}}\frac{W_{p}}{T}dt$$

Here, $t_{f}$ represents the time or the number of fatigue cycles at the point of fracture, T is the absolute temperature, and $W_{p}$ is the dissipated energy. Various methods for evaluating dissipated energy have been studied, such as measuring temperature changes and calculating plastic strain energy. Mehdizadeh et al. and Feng et al. [24,25,26] evaluated the fatigue fracture entropy (FFE) of metallic materials during multiaxial fatigue tests by measuring temperature using thermography and calculating dissipated energy from plastic strain. Their results showed good agreement between the number of cycles to fatigue failure and the fatigue life predicted from the FFE. Furthermore, Karimian et al. and Mahmoudi et al. [27,28] evaluated mechanical entropy generation from strain distribution during crack propagation and demonstrated that cracking occurs when entropy reaches a certain threshold. Many studies have applied entropy generation to the fracture criterion for metallic materials [23,24,25,26,27,28,29,30,31,32], polymeric materials [33,34,35], and composite materials [35,36,37,38].

In the past few years, studies have been conducted to evaluate entropy generation through thermal analysis [33,34,35]. Sakai et al. [35] assessed thermodynamic entropy generation by measuring the specific heat capacity of PA6 subjected to tensile deformation and found that thermodynamic entropy increases with volumetric strain. However, the free volume change, which is related to the increase in entropy [17], has not been experimentally evaluated. Furthermore, since most research has focused on thermoplastic resins, which have easily alterable internal structures, further studies on epoxy resin, a thermosetting resin, are essential for applications in structural components. The purpose of this study is to experimentally clarify the relationship between thermodynamic entropy generation and the free volume changes accompanying the tensile deformation of epoxy resin, a cross-linked thermosetting resin.

## 2. Materials and Experimental Procedure

### 2.1. Test Specimen Preparation and Dimensions

**Figure 1:**
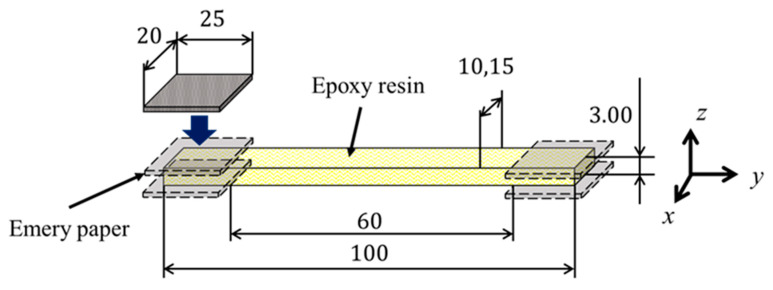
Dimensions of specimen of epoxy resin.

The epoxy resin was diglycidyl ether of bisphenol A (DGEBA), which was cured with 4,4′-diaminodiphenyl sulfone (4,4′-DDS). The epoxy (50.0 g) and amine (16.3 g) were stirred at 100 °C for 10 min and degassed at 80 °C. This procedure was repeated four times. The mixture was then poured into a mold and cured at 180 °C for 2 h. The resulting epoxy resin plate was cut into rectangular test pieces with dimensions of 100 mm in length, 10 mm in width, and 3.0 mm in thickness (Figure 1). To remove the load history, the pieces were heat-treated at 130 °C for 30 min. Simple tabs were attached to both ends of the test pieces using sandpaper. The width of the test pieces used for positron annihilation lifetime measurement was set to 15 mm to accommodate the size of the positron source.

### 2.2. Tensile Test and Digital Image Correlation Method

Tensile tests were conducted using a universal testing machine (AG-X Plus 50 kN, Shimadzu Corporation, Kyoto, Japan) at a tensile speed of 0.5 mm/min at room temperature (25 °C). Volumetric strain was measured using the digital image correlation (DIC) method. The strain distribution during tensile deformation was captured with a camera (L-836, HOZAN, Osaka, Japan), and DIC analysis was performed using DIC software (INSPECT Correlate 2023, Carl Zeiss AG, Oberkochen, Germany). The subset size was 19 × 19 pixels, and the point distance was 10 pixels. Poisson’s ratio (ν) was calculated from the average longitudinal strain ($\varepsilon_{y}$) and average transverse strain ($\varepsilon_{x}$) obtained from the DIC analysis. Assuming isotropic material behavior, the average strain in the thickness direction ($\varepsilon_{z}$) was calculated. Volumetric strain ($\varepsilon_{v}$) was then determined from the strains in the three orthogonal directions.

(2) $$\nu =-\frac{\varepsilon_{x}}{\varepsilon_{y}}$$

(3) $$\varepsilon_{z}=-\nu \cdot \varepsilon_{y}$$

(4) $$V_{2}=\left(x_{1}+\Delta x\right)\left(y_{1}+\Delta y\right)\left(z_{1}+\Delta z\right)$$

(5) $$\varepsilon_{v}=\frac{\Delta V}{V_{1}}=\frac{V_{2}-V_{1}}{V_{1}}=\left(\varepsilon_{y}+1\right)\left(\varepsilon_{x}+2\right)\varepsilon_{x}+\varepsilon_{y}$$

Here, *x*, *y*, *z*, and *V* represent the width, length, thickness, and volume, respectively, the subscript 1 represents the state before tensile deformation, and the subscript 2 represents the state after tensile deformation.

### 2.3. Differential Scanning Calorimetry (DSC)

To investigate the thermodynamic entropy of epoxy resin due to tensile deformation, specific heat capacity measurements were performed following the method used in a previous study [33,34,35]. A differential scanning calorimeter (DSC-60 Plus, Shimadzu Corporation, Kyoto, Japan) was used for specific heat capacity measurements. The samples for DSC measurements were scraped from the specimens before and after the tensile tests. Then, 3.00 mg of each sample was placed in a crimped aluminum pan and sealed with a crimp sealer. Alumina powder (α-Al_2_O_3_), 2.00 mg, was used as the calibration standard, with the samples prepared using the same procedure as that used for the measurement samples.

DSC measurements were conducted in a nitrogen atmosphere using a temperature modulation program. The temperature was increased at a rate of 2 °C/min, with a measurement temperature range of 0–80 °C, a modulation period of 60 s, and an amplitude of 0.3 °C. The change in heat flow between the measurement sample and the reference sample in the DSC experiment is expressed as follows:

(6) $$\frac{dQ}{dt}=C_{p}\beta +f\left(T_{DSC},t_{DSC}\right)$$

Here, dQ/dt is the heat flow obtained from DSC measurement, $C_{p}$ is the heat capacity, β is the temperature rate, and $f\left(T_{DSC},t_{DSC}\right)$ represents the heat flow resulting from the kinetic process, where $T_{DSC}$ and $t_{DSC}$ are the temperature amplitude and the temperature modulation period, respectively. In this study, the specific heat capacity $c_{p}$ was calculated by dividing $C_{p}$ by the weight of the epoxy resin.

**Figure 2:**
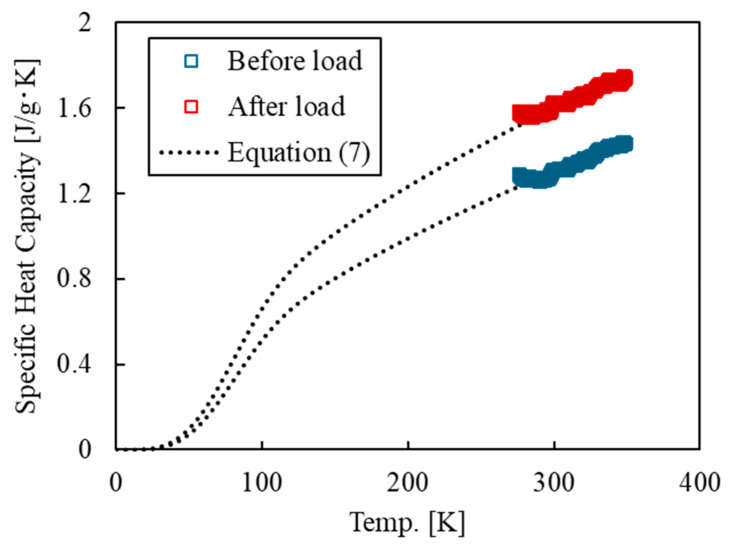
DSC results and fitting curve based on the Debye approximation [34] of the epoxy specimen before and after tensile tests.

To calculate the thermodynamic entropy $S_{T}$ from the specific heat capacity, the relationship between specific heat capacity and temperature from near absolute zero is required. Therefore, the DSC measurement results were extrapolated to 0 K using a specific heat capacity approximation equation based on the Debye model proposed by Kudo et al. [34] (Figure 2).

(7) $$c_{ps}=A\times T^{3}exp\left(-{\left(\frac{T}{T_{0}}\right)}^{n}\right)+\left(a\sqrt{T}+b\right)\left(1-exp\left(-{\left(\frac{T}{T_{0}}\right)}^{n}\right)\right)$$

Here, *A*, *a*, *b*, and n are fitting parameters, and $T_{0}\left(=80\;{}^{\circ}C\right)$ is the reference temperature. The thermodynamic entropy generation, $\Delta s_{T}$, due to tensile deformation can be calculated by subtracting the thermodynamic entropy in the unloaded state (strain = 0%) from the thermodynamic entropy calculated from the specific heat capacity obtained from the DSC measurement results of the test piece subjected to tensile deformation. Therefore, thermodynamic entropy generation, $\Delta s_{T}$, is calculated using the following equation:

(8) $$\Delta s_{T}=\int_{T_{1}}^{T_{2}}\frac{\Delta c_{ps}}{T}dT$$

Here, $T_{1}$ indicates the starting temperature of the DSC measurement, and $T_{2}$ indicates the target temperature of the DSC measurement.

### 2.4. Positron Annihilation Lifetime Spectroscopy (PALS)

Positron annihilation lifetime spectroscopy (PALS) was performed using a Positron Surface Analyzer (Toyo Seiko Co., Ltd., Aichi, Japan, Positron Surface Analyzer Type L-P) with a 22 Na (~1 MBq) positron source. The total count of the positron lifetime histogram was 1.0 × 10^6^ counts, measured at room temperature (25°C). The obtained positron lifetime histogram was fitted using positron annihilation lifetime analysis software (Toyo Seiko Co., IPALM Ver. 2.4.3). From the fitting curve, the positron annihilation lifetime was decomposed into three components, *τ*_1_, *τ*_2_, and *τ*_3_ (*τ*_1_ < *τ*_2_ < *τ*_3_), and the average lifetime [ns] and relative intensities [%] (*I*_1_, *I*_2_, *I*_3_; *I*_1_ + *I*_2_ + *I*_3_ = 100%) for each component were calculated. The components *τ*_1_, *τ*_2_, and *τ*_3_ correspond to the annihilation lifetimes of para-positronium (p-Ps), a free positron, and ortho-positronium (o-Ps), respectively. In this study, the most significant lifetime component is *τ*_3_, which corresponds to the annihilation of o-Ps in the free volume of polymer materials [39]. Therefore, the first two lifetime components, *τ*_1_ and *τ*_2_, are not discussed further.

**Figure 3:**
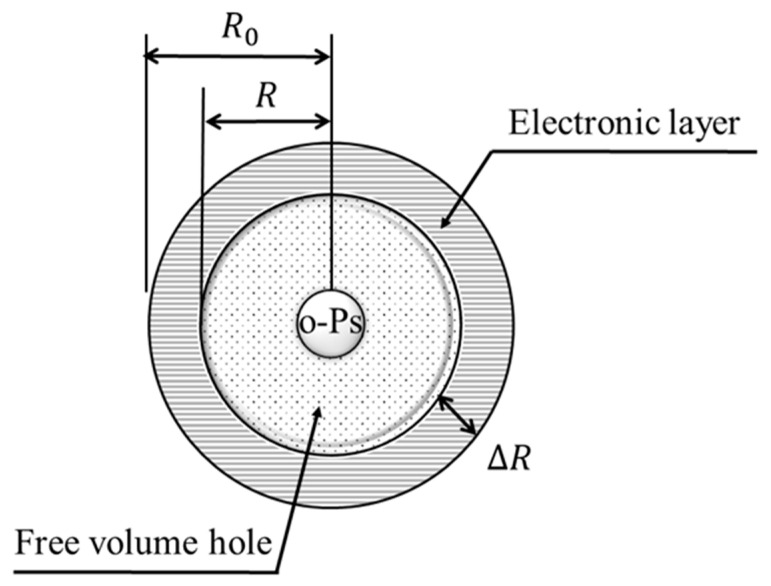
Schematic diagram of free volume radius in Tao–Eldrup model.

The positron lifetime component *τ_3_* was converted to the free volume radius using Equation (9) and the Tao–Eldrup model, as shown in Figure 3. Furthermore, the measurement data were fitted to the model Equation (10), which assumes a log-normal distribution of free volume sizes in the sample, using least squares fitting. From this, the lifetime distribution and free volume radius distribution were calculated, along with the corresponding average and standard deviation.

(9) $$\tau_{3}=0.5{\left(1-\frac{R}{R_{0}}+\frac{1}{2\pi }\sin\frac{2\pi R}{R_{0}}\right)}^{-1}$$

(10) $$g_{l}\left(t\right)=\int_{0}^{\infty }g\left(t;\tau ,T_{0}\right)l\left(\tau ;\mu ,\sigma \right)d\tau$$

(11) $$g\left(t;\tau \right)=A\;\exp\left(-\frac{t}{\tau }\right)$$

(12) $$l\left(v;\mu ,\sigma \right)=\frac{1}{\sqrt{2}\pi \sigma v}exp\left(-\frac{{\left(\ln v-\mu \right)}^{2}}{2\sigma^{2}}\right)$$

Here, *R* is the free volume radius, ΔR is the electron layer thickness (=0.1666 nm), $R_{0}=R+\Delta R$, τ is the positron lifetime, and $g\left(t;\tau \right)$ is the decay function that represents the histogram of positron lifetimes annihilated at the free volume radius corresponding to τ. $l\left(v;\mu ,\sigma \right)$ is the logarithmic distribution of a random variable following a normal distribution with a mean μ and variance $\sigma^{2}$. To investigate the change in free volume due to tensile deformation, PALS measurements were performed on the test specimens both before tensile deformation and after tensile fracture.

## 3. Results and Discussion

### 3.1. Results of Tensile Test

**Figure 4:**
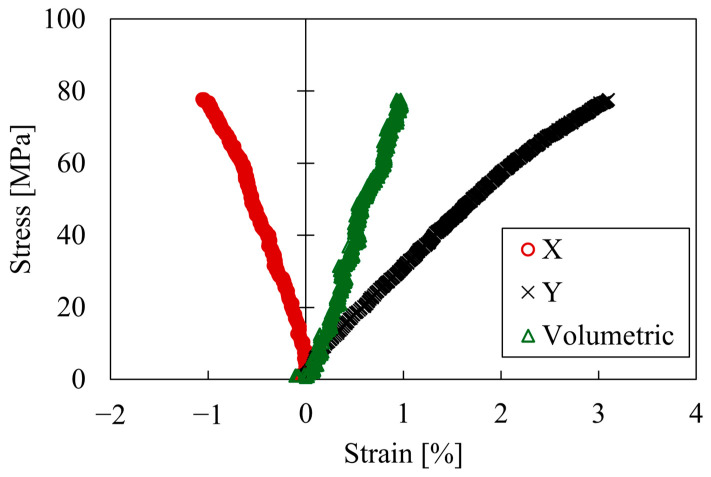
Stress–strain–volumetric curves of epoxy.

Figure 4 shows a typical stress–strain curve obtained from the tensile test. The vertical axis represents nominal stress. The horizontal axis represents the strain obtained from the DIC results, and X and Y represent the horizontal and vertical strains, respectively. The average Poisson’s ratio calculated from the X and Y strains and Equation (2) was 0.321. The volumetric strain was calculated by substituting the X and Y strains and the Poisson’s ratio into Equations (3)–(5). The longitudinal strain increased linearly up to a strain of 2%, after which the behavior changed. The volumetric strain increased linearly with deformation. The relationship between the longitudinal strain and stress does not show a yield point, so the epoxy resin is considered to fracture due to its brittle nature. The absence of a yield point is due to the cross-linked structure of epoxy resin.

### 3.2. Thermodynamic Entropy Generation of Epoxy Resin with Deformation

**Figure 5:**
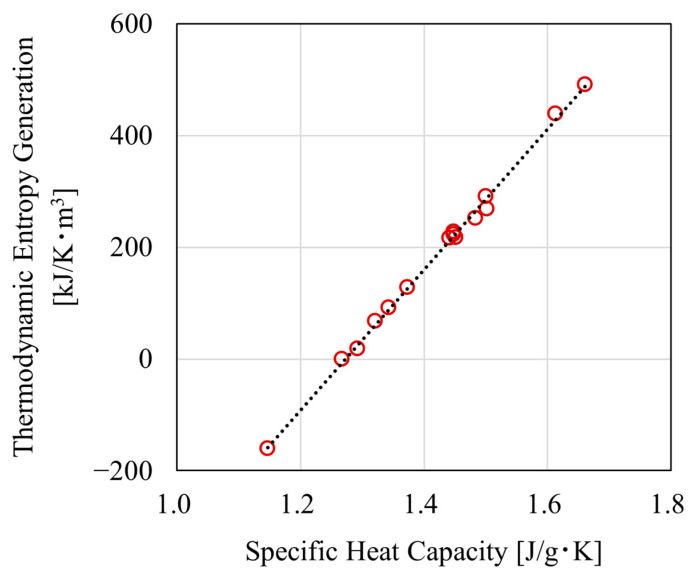
Relationship between specific heat capacity and thermodynamic entropy generation at 25 °C.

**Figure 6:**
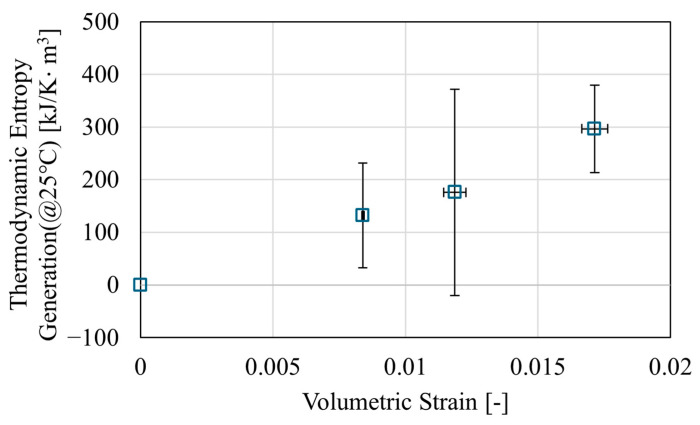
Relationship between volumetric strain and thermodynamic entropy generation.

Before the relationship between deformation and thermodynamic entropy generation can be compared, it is necessary to clarify the relationship between the measured specific heat capacity and thermodynamic entropy generation. Figure 5 shows the relationship between specific heat capacity and thermodynamic entropy generation at 25 °C. The horizontal axis represents the specific heat capacity measured at 25 °C, obtained from DSC measurements, while the vertical axis shows thermodynamic entropy generation, calculated from Equations (7) and (8). The fitted line in Figure 5 was calculated using the least squares method based on the relationship between the specific heat capacity measurement results and thermodynamic entropy generation. In Figure 5, the specific heat capacity and thermodynamic entropy generation show a linear relationship. Although the specific heat capacity may change at high strain, the linear relationship between the specific heat capacity and thermodynamic entropy generation is expected to be maintained even at high strain. This is because thermodynamic entropy generation is determined by the specific heat capacity and the measurement temperature range of the DSC, as shown in Equation (8). This suggests that thermodynamic entropy generation can be evaluated through specific heat capacity measurements. From here onward, we will focus on changes in entropy generation. Figure 6 shows the relationship between volumetric strain and thermodynamic entropy generation. The vertical axis represents the thermodynamic entropy generation at 25 °C, and the horizontal axis represents the volumetric strain. From these results, it was confirmed that thermodynamic entropy generation tends to increase linearly with volumetric strain, although some variation was observed. Therefore, it was demonstrated that thermodynamic entropy is generated by tensile deformation even in epoxy resin, a thermosetting resin with a cross-linked structure. It was also suggested that this thermodynamic entropy generation may be related to the volume increase caused by deformation, as thermodynamic entropy was observed to increase with increasing volumetric strain.

### 3.3. PALS Measurement Results

**Figure 7:**
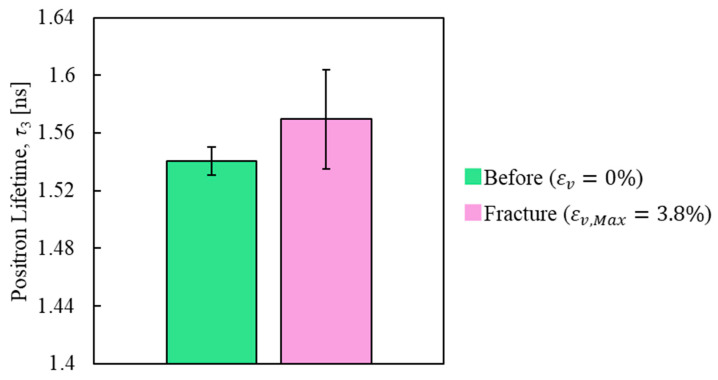
Positron lifetime under no load and after tensile fracture.

**Figure 8:**
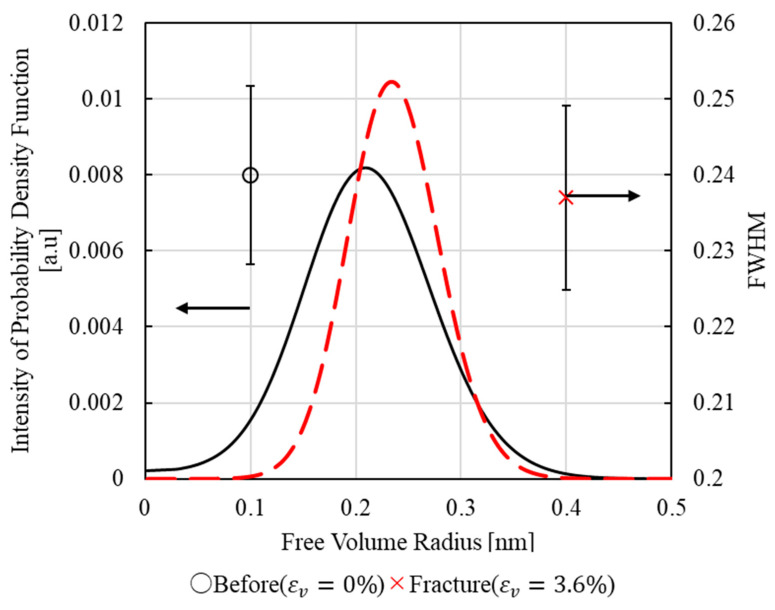
Free volume radius distribution and FWHM change due to tensile tests.

Figure 7 shows the positron annihilation lifetime component τ_3_ obtained from the results of PALS measurements performed before and after the tensile test. The results show that the lifetime component τ_3_ increased after tensile deformation, which is likely related to the structural change in the epoxy due to tensile fracture [21]. To quantitatively evaluate the change in the free volume size, the lifetime component τ_3_ was converted to the free volume radius using Equation (9). Figure 8 shows the free volume radius on the horizontal axis, the intensity of the probability density function on the left vertical axis, and the full width at half maximum (FWHM) on the right vertical axis. In Figure 8, the free volume radius of the epoxy resin is concentrated in the range of approximately 0.2 to 0.3 nm. It was observed that the peak value of the radius distribution increased, while the FWHM tended to decrease, after tensile deformation. These findings suggest that tensile deformation may increase the free volume radius and make its distribution more uniform.

To evaluate the volume change, we defined the total free volume $V_{Total}$ as shown in Equations (13) and (14) based on the *i*-th average radius $R_{i}$ and the probability density $I_{i}$ of $R_{i}$ in the radius distribution (Figure 8).

(13) $$V_{Total}=\overset{n}{\underset{i=0}{\sum }}V_{i}\cdot I_{i}$$

(14) $$V_{i}=\frac{4}{3}\pi {R_{i}}^{3}$$

**Figure 9:**
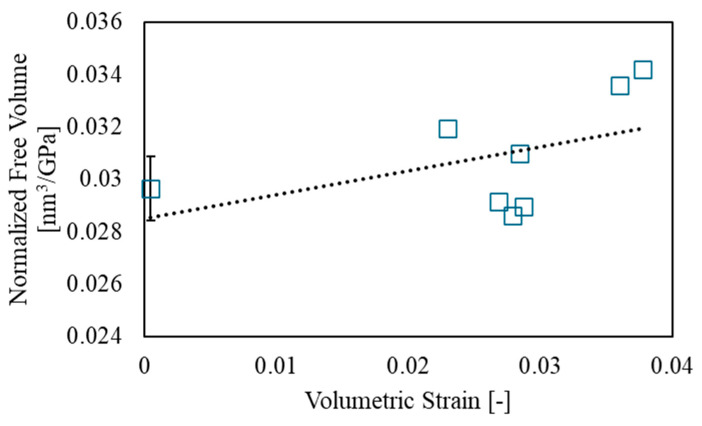
Relationship between volumetric strain and total free volume.

It is well established that the free volume of polymer materials varies with their Young’s modulus [14,15,40]. To account for material-dependent variability in this study, the total free volume, $V_{Total}$, was normalized by the Young’s modulus (in GPa) obtained from individual tensile tests. Figure 9 shows the relationship between volumetric strain and the normalized total free volume. From this relationship, it was found that free volume shows a slight positive increase with an increase in volumetric strain, although some variation was observed. The following equation, which describes the relationship between volumetric strain and free volume, was obtained.

(15) $$V_{Total,Norm}=c\varepsilon_{v}+d$$

Here, c and d are material constants, and $\varepsilon_{v}$ is the volumetric strain. The material constants were calculated using the least squares method, yielding c=0.0895 and d=0.0286. From the above-mentioned methods, it has been experimentally demonstrated that the free volume of epoxy resin tends to increase with tensile deformation. This nanoscale change is thought to have increased the material’s heat storage capacity by expanding its internal area, possibly leading to an increase in thermodynamic entropy.

### 3.4. Relationship Between Thermodynamic Entropy Generation and Free Volume

**Figure 10:**
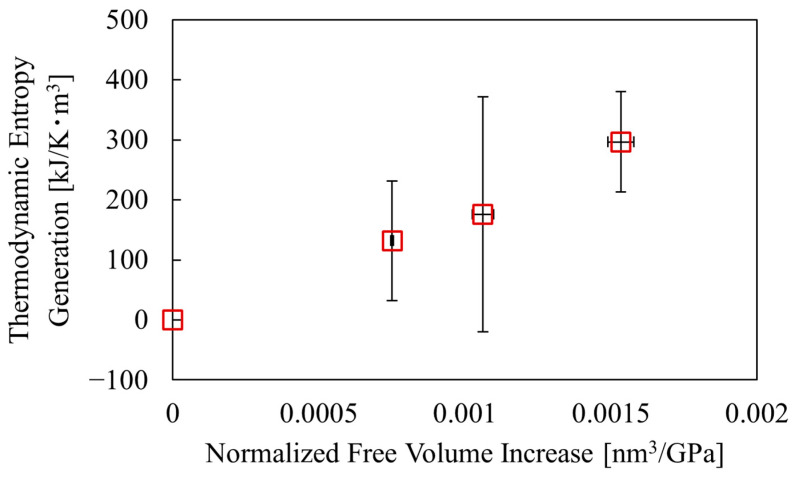
Relationship between free volume increase and thermodynamic entropy generation.

Figure 10 shows the relationship between the increase in free volume and the thermodynamic entropy generation calculated using Equation (15). Although there is some scatter in the results, there is a tendency for thermodynamic entropy generation to increase with an increase in free volume. This supports the idea that the free volume and thermodynamic entropy of polymeric materials tend to increase with tensile deformation, as in the results of MD simulations [17], suggesting that there may be a linear relationship between them.

Based on the above-mentioned findings, it is suggested that the thermodynamic entropy generation observed during the tensile deformation of epoxy resin may result from irreversible changes, such as an increase in the material’s internal free volume.

### 3.5. Internal Changes in Materials During Entropy Generation Processes

**Figure 11:**
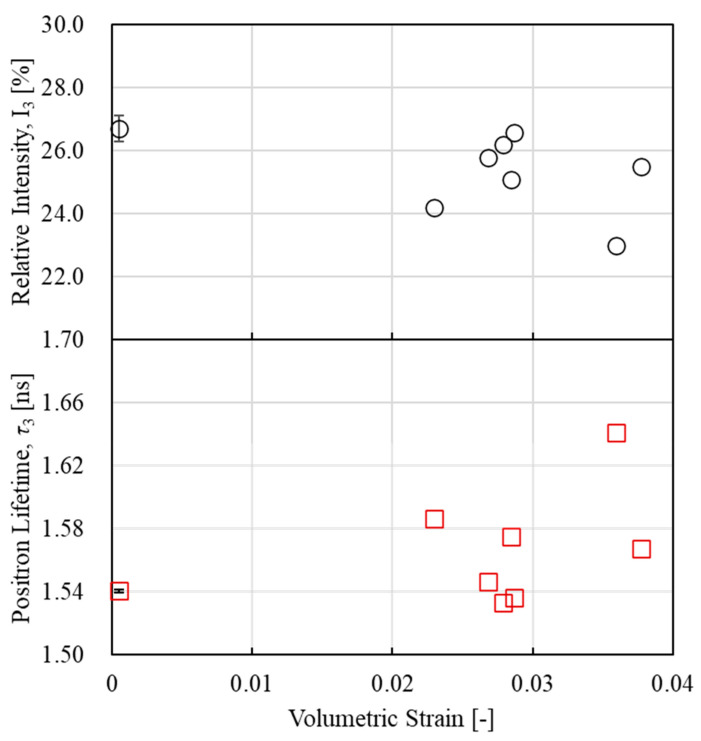
Relationship between volumetric strain and positron annihilation lifetime component $\tau_{3}$ and relative intensity $I_{3}$.

**Figure 12:**
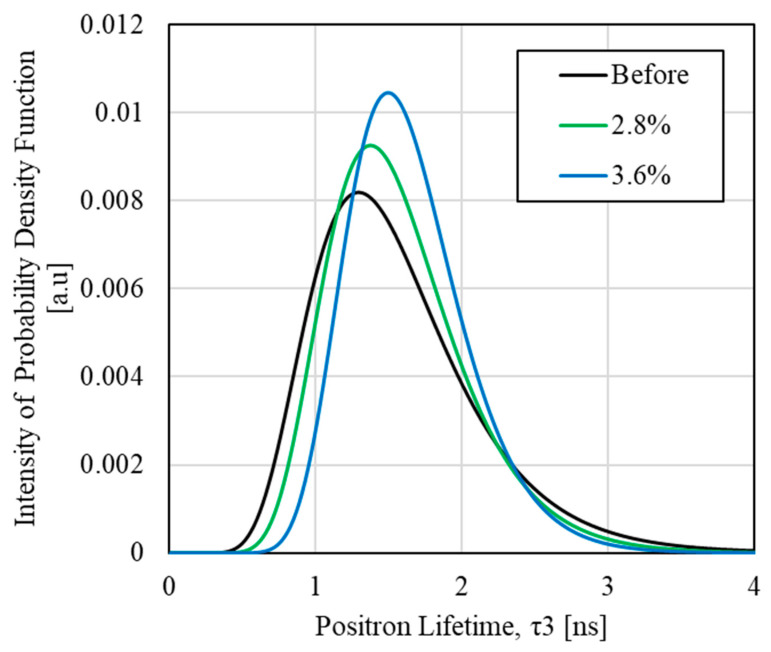
Distribution of positron annihilation lifetime component $\tau_{3}$ at no load and after fracture (volumetric strain of 2.8 and 3.6%).

To evaluate the internal changes in the material during the entropy generation process of epoxy resin, we focused on the positron lifetime component $\tau_{3}$ and its relative intensity $I_{3}$, obtained from PALS measurements. Figure 11 shows the relationship between the lifetime component $\tau_{3}$, its relative intensity $I_{3}$, and volumetric strain. These results show that the life component τ_3_ shows an overall increasing trend with increasing volumetric strain, while the relative intensity $I_{3}$ shows a slight decrease in the region below 3% volumetric strain and a decrease in the region above 3% volumetric strain. Uematsu et al. [41] showed that when the lifetime component $\tau_{3}$ increases and its relative intensity remains constant, the change in the positron lifetime is mainly governed by the change in the size of free volume. In contrast, in regions where the relative intensity decreases, small free volumes may merge. We focused on a volumetric strain of 3%, where the tendency for the relative intensity to decrease changed. Therefore, to compare the positron lifetime distributions for volumetric strains below 3% and at or above 3%, Figure 12 shows the positron annihilation lifetime distributions for volumetric strains of 2.8% and 3.6%. For comparison, the distribution of the positron annihilation lifetime for an unloaded sample is also shown. The horizontal axis of Figure 12 represents the positron annihilation lifetime component *τ*_3_, while the vertical axis represents the intensity of the probability density function. As shown in Figure 12, there is little difference in the change in the peak position with respect to unloading at volumetric strains below 3% and at or above 3%. However, the increase in peak intensity at a strain of 3.6% is more significant compared to that at 2.8%, which may indicate a change in the distribution of positron annihilation lifetimes around the 3% strain threshold. Therefore, it has been suggested that molecular chain breakage may occur at a volumetric strain threshold of 3%.

**Figure 13:**
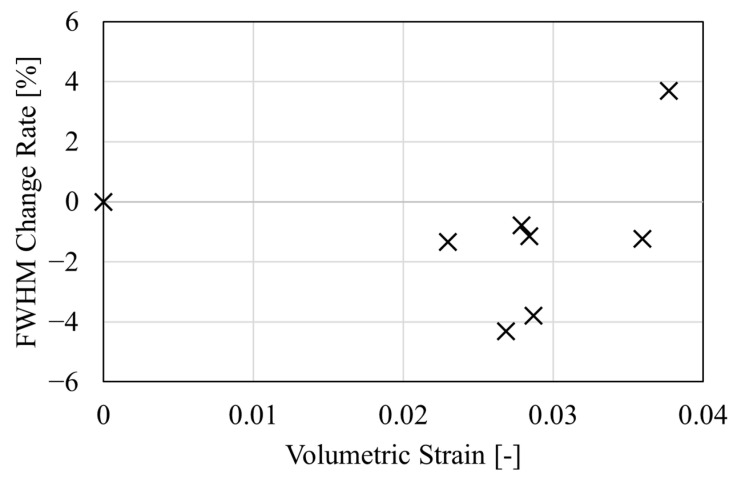
Relationship between volumetric strain and full width at half maximum change rate.

Figure 13 shows the relationship between the reduction rate of the full width at half maximum (FWHM) of the positron annihilation lifetime distribution and volumetric strain. The vertical axis represents the reduction rate of FWHM, with the unloaded FWHM normalized to 100%. The horizontal axis represents the volumetric strain. As shown in Figure 13, there is a slight decrease in FWHM following tensile deformation. This change may be attributed to free volume becoming more uniform because of tensile deformation. In these results, the increase in FWHM at one point at a volumetric strain of 3.8% is thought to be due to measurement variance. To better understand the change in the FWHM of the positron annihilation lifetime due to deformation, further investigations with an increased number of tests are needed in future studies. The above-mentioned findings suggest that during the entropy generation process of epoxy resin, free volume may increase due to changes in the size of free volume inside the material and the coalescence of small free volumes.

Therefore, it is suggested that the thermodynamic entropy of epoxy resin increases with tensile deformation and that entropy generation may be related to an increase in free volume.

## 4. Conclusions

The relationship between thermodynamic entropy generation and the change in free volume accompanying the tensile deformation of an epoxy resin composed of DGEBA and 4,4′-DDS was investigated. It was found that thermodynamic entropy generation, calculated from specific heat measurements, exhibited an increasing trend with increasing volumetric strain. Both the free volume and its average radius also showed an increasing trend with increasing volumetric strain. Moreover, the peak value of the free volume radius distribution increased, while the full width at half maximum (FWHM) decreased. Thermodynamic entropy generation demonstrated a linear increasing trend with the increase in free volume. This finding suggests that the increase in thermodynamic entropy accompanying tensile deformation may be associated with irreversible changes, such as an increase in free volume. Clarifying the relationship between irreversible changes, such as thermodynamic entropy generation, and the expansion of free volume may be useful in developing fracture criteria for thermosetting resins, particularly by linking fracture to the possibility of crack formation when free volume expands to a certain size. These results could contribute to research into applying entropy generation as a fracture criterion, a topic of increasing interest in recent years.

## Data Availability

The original contributions presented in this study are included in the article. Further inquiries can be directed to the corresponding author.
